# Live cell imaging of exogenous α-synuclein fibrils in primary microglia and neuron co-cultures

**DOI:** 10.1016/j.bbrep.2025.102416

**Published:** 2026-01-06

**Authors:** C. Paquette, T. Charlton, N. Prowse, T. Fortin, H. Sun, S. Hayley

**Affiliations:** Department of Neuroscience, Carleton University, 1125 Colonel By Drive, Ottawa, Ontario, K1S 5B6, Canada

**Keywords:** Parkinson's, A-synuclein, Inflammatory, Microglia, Cytokine, Co-culture

## Abstract

α-synuclein (α-syn) rich Lewy bodies are a prominent pathological feature of Parkinson's disease (PD), with intra-cellular accumulation occurring in neurons and possibly microglia. Tracking α-syn movement between the two different cell types is of critical importance in determining how pathology spreads. We hypothesized that the separate pre-treatment of either primary cortical neurons or microglia with exogenous α-syn preformed fibrils (PFFs) will foster a cytotoxic environment when co-cultured with the opposite naïve cell type. To this end, using real time live cell imaging, we found an accumulation of Alexa Fluor 488 labelled α-syn PFFs in both microglia and neurons. In the co-cultures, the labelled-PFFs showed differing patterns of spread to non-seeded cells. The PFF treatment also provoked cellular loss that increased with the passage of time and induced marked vacuolation and changes in microglial morphology. Microglia appeared to accumulate PFFs from morphologically compromised neurons and shifted to a predominately dystrophic and “foamy enlarged fried egg” morphology over time and was associated with a reduction in levels of the anti-inflammatory cytokine, interleukin-4 (IL-4). We currently provide a novel *in vitro* co-culture model that allows for tracking α−syn spread between primary cortical microglia and neurons.

## Highlights

• A novel primary microglia-neuron co-culture system for imaging a-synuclein fibrils.
• Selective a-synuclein seeding of neuronal vs microglial promotes dramatic cellular interactions.
• a-synuclein fibrils caused time-dependent neurotoxic and gliotoxic effects.
• a-synuclein fibrils promote microglial phenotypic shifts.

## Graphical abstract

**Figure:**
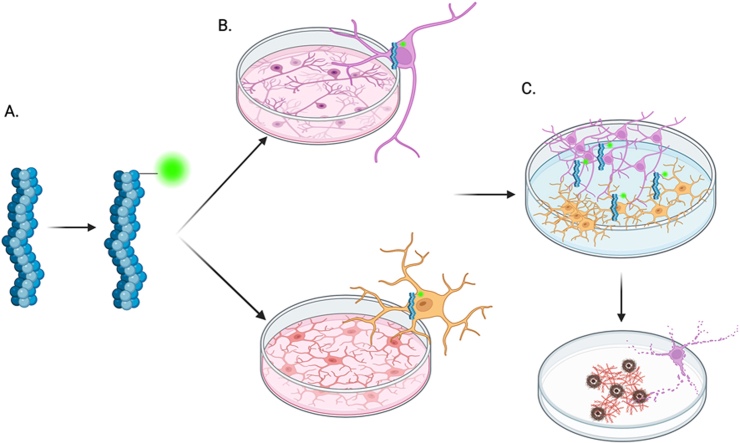

## 1 Introduction

Two primary neuropathological hallmarks of Parkinson's disease (PD) are the loss of dopaminergic (DAergic) neurons of the nigrostriatal system and the development of intracytoplasmic Lewy bodies that are rich in aggregated and misfolded α-synuclein (α-syn) [1,2]. The α-syn protein exists in both monomeric and oligomeric states [3], but the protein can also adopt a potentially toxic oligomeric or a β-sheet fibril conformation [4]. Many studies have now utilized α-syn pre-formed fibrils (PFFs) to induce α-syn aggregates and Lewy-like pathology [5,6].

Microglia are highly responsive to α-syn perturbations [[7], [8], [9], [10]], with aggregates inducing neuroinflammatory responses [3,11]. Yet, anti-inflammatory microglia can foster tissue repair, extracellular matrix reconstruction and may help clear pathological α-syn by phagocytosis [11,12]. The morphology of microglia varies between the differing phenotypic states and can be used as a proxy for degree/type of activation [[13], [14], [15]]. Real-time imaging is an invaluable tool for assessment of microglia behavior and interaction with co-cultured neurons.

The present study sought to assess the impact of exogenously applied α-syn PFFs upon primary neuron and microglia co-cultures. It was of interest to selectively pre-treat neurons or microglia with PFFs and then co-culture the treated cells with the untreated opposite cell type. By using this co-culture system with real-time imaging technology, we found that PFFs can directly accumulate in both microglia and neurons and then induce time-dependent loss of PFF + cellular area. We also found that PFFs induced marked morphological changes in microglia and a reduction in interleukin-4 (IL-4) levels in the co-cultures. This work shows the dynamic spread of PFFs in microglia and neurons and has relevance for understating time and cell-dependent processes that might be operative in PD and α-synucleinopathies in general.

## 2 Methods

### 2.1 Animals

C57BL/6 mice (Charles River Laboratories) were bred to produce embryonic (E) and post-natal (P) litters for *in vitro* cultures. Animals were harem mated (1 male to 2 females), given ab libitum access to water and lab chow and were left on a normal 12-h light cycle. For embryonic neuronal cultures, the male was removed 48 h after initial mating and the females remained together. For post-natal glial cultures, the male was removed two weeks following initial mating and the females were separated. Multiple breeders were produced to provide a minimum of 8–10 animals per experiment including both female and male animals. All aspects of this experiment were approved by the Carleton University Committee for Animal Care and adhered to the CCAC ethical standards (ethics approval number AUP 121415).

Animals were euthanized by rapid decapitation and cortical neuronal cultures were collected at E16-17, as this is a critical period for cortical development. During this time, the generation of pyramidal neurons is almost complete and there are few glial cells present. In contrast, mixed glial cultures were collected at P1-3 to ensure easy dissection and a high yield of viable glia. Both cell cultures were obtained from the cortex, as this area typically displays a particularly high concentration of aggregated α-syn in the PD brain. We had a biological n = 3/group (i.e. 3 different cultures from three different dams) with a technical n = 5 replications over wells.

### 2.2 Primary neuron cell culture

Cortical neuronal tissue was collected using adapted protocol established by Hilgenberg and Smith [16] and Gaven et al. [17], wherein primary neurons were extracted from the cortex and mild trypsinization (with TrypLE) applied. They were then plated on Poly-d-Lysine (PDL) (10μg/mL in Dulbecco's Modified Eagle Media (DMEM)) coated HNO_3_ etched coverslips in 24-well plates at a concentration of 1 × 10^5^ cells/mL. Cultures were maintained in complete neurobasal media (CNB: 97.25 % Neurobasal media, 2 % B27 supplement, 0.5 % Penicillin/Streptomycin (pen-strep), 0.25 % GlutaMAX [all Gibco]) at 37 °C in a 90 % humidified air and 5 % CO_2_ incubator. A full media change was performed the day after surgery to remove debris, using new CNB without GlutaMAX and further half media changes occurred every 2–3 days. Neuron cultures were utilized for experiments on DIV8-10 since the neurons appear well developed with connected with extensive networks at this time and are still plastic enough to maximize survival.

### 2.3 Primary microglia cell culture

Collection of primary microglia (Saura et al., 2003; Schildge et al., 2013), first involved the extraction of mixed glial tissue from the cortex. The tissue was then trypsinized (1:1:1 of 0.25 % Trypsin, DMEM-F12 and Versene), plated in T75 flasks (2–3 cortices/flask), coated with PDL (10μg/mL) and maintained in complete media (89 % DMEM, 10 % heat-inactivated fetal bovine serum (FBS), 1 % pen-strep) at 37 °C in a 90 % humidified air and 5 % CO_2_ incubator. A full media change was performed the day after surgery to remove debris, and half media changes occurred every 4–5 days thereafter. The mixed glial culture first develops an astrocyte monolayer, with microglia appearing once confluency is reached. The astrocyte monolayer allows for the continued growth and proliferation of the microglia until a sufficient yield can be obtained for experimental use. The primary microglia were then isolated from the mixed-glial cultures at DIV 22–25, which we found optimal to yield mature purified microglia. The absence of astrocytes was verified by a lack of GFAP + cells and the specific morphological aspects of cell present in the culture.

### 2.4 Preparation of α-syn PFFs

1 mg of human α-syn monomer protein (Proteos, cat. RP003) was used to create the PFFs over seven days. Aliquots of α-syn monomer were thawed for 3 h on ice then centrifuged (4 °C; 112×*g*). The supernatant was moved to a 1.5 mL autoclaved microcentrifuge tube and concentration was determined by a NanoDrop 2000 spectrophotometer using the A280 protein method. A 2 μL blank of 10X Dulbecco's Phosphate Buffered Saline (DPBS) and 2 μL of sample were used and concentration was measured using Beer's law (ɛ = 5960; kDa – 14.6). The PFFs were the diluted in 10X DPBS for a final concentration of 5 mg/mL and briefly vortexed. The tubes were then added to an Eppendorf Thermomixer R at 37 °C and shaken for 7 days at 112×*g*. PFFs were aliquoted into 25 μL samples, frozen on dry ice and stored at −80 °C until use.

In order to label the α-syn PFFs, they were first sonicated in 1 mL with one pulse every 2 s for 60 s. Then they were labelled using an Invitrogen Alexa Fluor 488 protein labelling kit (A10235).

### 2.5 Experimental treatments

**Fig. 1:**
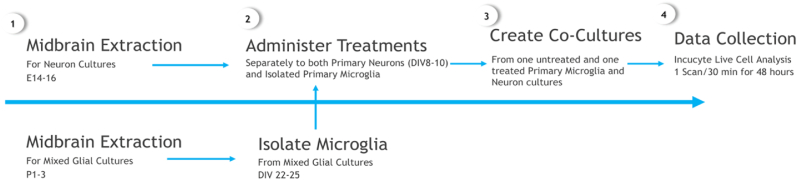
**Primary Co-Culture Timeline. (1)** Cortical tissue was extracted from the pups on post-natal Day 1–3 (P1-3) for the mixed glial cultures and from embryos on embryonic day 16 (E16) for neuron cultures. **(2)** After 22–25 days of *in vitro* proliferation (DIV 22–25), primary microglia were isolated and extracted from the mixed-glial cultures. On the same day, the isolated microglia and neuronal cultures were treated with 488 labelled α-syn PFFs or were left untreated (controls). **(3)** The primary microglia-neuron co-cultures were then created by adding untreated microglia directly to established treated primary neuron cultures or conversely, treated microglia was added directly to established untreated neuron cultures. **(4)** The co-cultures were then immediately placed in an Incucyte live cell analysis instrument and were imaged every 30 min for 48 h in total. Following this, media was collected from each co-culture for cytokine analyses.

Fig. 1 outlines the timeline of experimental manipulations. After 8–10 (for neurons) or 22–25 (for microglia) days *in vitro,* the Alexa Fluor 488 labelled PFFs were administered. Separately cultured primary neurons or isolated microglia were either left untreated or exposed to 488-labelled PFFs (2.5 μg/mL in 4 mL of new media). The separate neuron or microglia cultures were treated for 24 h to allow for sufficient PFF uptake. At this point, the co-cultures were created by adding the individual treated microglia or neuronal cultures to the opposite cell type (treatment naïve). This involved the microglia being removed using methods similar to that of Woolf et al., 2021, which included firmly taping and using a sterile rubber cell to gently scrape the bottom of flask to cause microglial detachment. These cells were then immediately added to the established neuron cultures in 24 well-plates, such that each neuron-microglia co-culture had one treated cell type and one untreated cell type. This was performed with a full media change to ensure that only the previously administered PFFs that had incorporated within the cells (and not any that might be in the extracellular media) was present in the co-cultures. The co-cultures were then immediately placed in an Incucyte S3-Live cell analysis Instrument (Sartorius, CAT 4647) and videos recorded. This system allows real-time assessment of cell-cell interactions and changes in morphology and motility.

### 2.6 Image analysis and quantification

All image analyses were conducted by a rater blinded to the experimental conditions. All images were obtained directly from the Incucyte software in phase or Alexa 488 (green) uncalibrated format and analyzed using the FIJI image analysis software. Live video was collected for the entire 48-h period of the experiment, with 1 min of video captured at 30-min sampling intervals. Scale was set for all images at 1.61 pixels/μm with the entire image frame used in each analysis and set to 874.53 μm × 645.96 μm.

### 2.7 Determination of change in cellular coverage area

Phase Images at times 0h, 8h, 24h, 32h and 40h for the co-cultures were obtained for each treatment. The “Find Edges” function was used to highlight the cell structures within a particular frame. An appropriate thresholding method (Li) was used and the background was selected. “Analyze Particles” was then used to determine the percentage of background area within the frame. Subtracting this value by the total area of the frame leaves the percentage of cellular structures present. The percentage change (% Δ) of this value was calculated for each time interval (V_2_) in relation to what was found at time 0h (V_1_) to represent the percentage of change in cellular coverage area observed over time. Equation is as follows:

$$\%\;\Delta =\frac{V_{1}-V_{2}}{V_{1}}*100\%\text{.}$$

### 2.8 Fluorescent signal intensity and cellular morphological differences

The uncalibrated green (488 nm) images (Raw 16-bit) at times 0h, 8h, 16h, 24h, 32h, 40h and 48h were obtained from the experimental groups. The appropriate thresholding method (Triangle for PFFs) was used and dark background was selected. For the 488 Labelled PFF analysis, the range was set to 1.25 μm-infinity with the exclusion of signals on the edges of the frame and the inclusion of holes within the signal. “Analyze Particles” function was used to determine the Integrated Density (IntDen), a sum of all the pixels within the frame denoting the overall fluorescent intensity (product of the Mean gray value and the Area). The percentage of observed PFF intensity at 24h and 48h was calculated to determine the overall difference (increase/decrease) in their accumulation. The IntDen observed at 24h and 48h (V_2_) was compared to the IntDen at 0h (V_1_).

The % Δ was also calculated during the time intervals. IntDen of treatment groups containing 488 Labelled PFFs from all time points (V_2_) were compared to what was observed in the time point occurring directly before it (V_1_) (Ex. 0h–8h, 8h–16h ….40h–48h).

Individual PFF + cells were segmented using Analyze Particles function with ImageJ functions selected to automatically measure cell area, circularity and aspect ratio. We outlined five cells for each biological sample (n = 3), for each experimental condition. Cellular area essentially measures cell size, whereas for circularity, the formula is 4π(area/perimeter^2^), with a value approaching 1.0 indicating increasingly round morphology. Aspect ratio was derived from the ratio of the major to minor axes of the best-fit ellipse (essentially ration width to height), reflecting cellular elongation. Identical thresholding and segmentation parameters were applied across all cells.

### 2.9 Microglia morphology classification

Phase images at times 0h, 8h and 32h for were obtained in each treatment group. All microglia present within the frame were counted and classified manually into one of five groups based on observed morphology (1. Ameboid, 2. Rod, 3. Dystrophic or 4. Fried Egg). Ameboid microglia were defined as uniform, small diameter circular cells, lacking processes or having 0–2 short and thin processes with no vacuoles. Rod microglia (also called bipolar) were denoted by a central nucleus with two long and thin processes. Bushy microglia are characterized by an enlarged, circular cell body (in comparison to ameboid morphology) and short but thicker processes containing a few vacuoles (≈4–8 μm). Dystrophic microglia (also called hypertrophic) demonstrated varied reactive morphologies but overall were classified as cells with elongated cells bodies with multiple often highly branched processes typically containing multiple vacuoles (≈7–11 μm). Fried egg microglia were classified as lacking processes with large, flattened and expanded cell bodies with multiple vacuoles (+10). The percentage of each morphology within each frame was collected for all treatment groups.

### 2.10 ProQuantum immunoassay of cytokine levels

ProQuantum high sensitivity immunoassay kits (ThermoFisher) were used to measure the extracellular levels of TNF-α, IL-6 and IL-4. Media was collected from each treatment group immediately following the 48h of imaging and concentration was determined using the manufacturer's instructions.

### 2.11 Statistical analyses

All data was analyzed using the Prism (version 9) statistical software and is presented as the marginal mean ± standard error of the mean (mean ± SEM) from three biological replicates, from three different litters (each containing 5 technical replicates). Cytokine results were analyzed by cell treatment using a one-way ANOVA. The remainder of the data was analyzed by Treatment vs Time in a repeated measures two-way ANOVA and the Geisser and Greenhouse methods were used to correct for violations to the assumption of sphericity. All results with significant interactions were followed by multiple comparison with Tukey's test correction. Differences were considered as statistically significant when p < 0.05.

## 3 Results

### 3.1 Neurons and microglia show opposing changes in PFF fluorescence

We first sought to confirm that primary neurons and microglia can each individually take up and accumulate exogenously applied 488-labelled PFFs. To this end, we first utilized neuronal and microglial monocultures and found that both cell types easily accumulated the PFFs within hours.

**Fig. 2:**
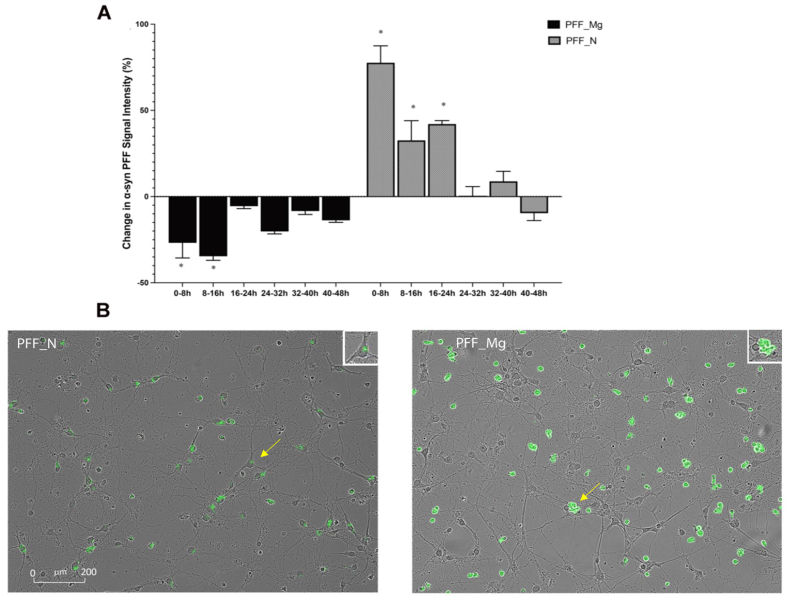
**Progressive shift in total frame fluorescence intensity (PFF-channel) over time. (A)** The time-dependent percentage change in 488 labelled α-syn PFF intensity in 8-h time intervals over 48 h is shown in the bar graph. PFF + labelling was initially (0 h in co-culture) greater in pre-treated microglia than neurons, but there was a rapid reduction in the PFF signal in microglia over time. Whereas the converse was true for pre-treated neurons, wherein there was a rapid increase followed by decline in the PFF signal, likely owing to cellular migratory changes followed by neuronal death. **(B)** Obvious PFF-488 labelling was observed in neurons and microglia at the beginning of co-culture (i.e. after their respective 24 h PFF incubation times alone, at time 0 h when co-cultures were created). Co-cultures at the initial time point (0 h) from PFF pre-treated neurons (PFF_N) or microglia (PFF–Mg) are shown in photomicrographs, respectively. The inset (top right and indicated by arrow) in PFF_N shows an example of cytoplasmic PFF labelling in a neuron and the inset in PFF_Mg depicts an example of several round-ameboid microglia clustered together around neurons [N = 3 biological replicates: (i.e. cells from separate litters). and 3–6 technical replicates (i.e. from different wells per condition/litter).] Graph shows mean ± SEM.

After confirming PFF uptake in microglial and neuronal cells, we next created co-cultures (Fig. 1) in order to assess PFF cell specific effects. Specifically, we assessed PFF seeded microglia interactions with naïve neurons and vice versa, how PFF seeded neurons would interact with naïve microglia. Analysis of the percentage change in 488-PFF intensity, in 8-hr bins, was used to measure the fluctuations in total frame fluorescence intensity (α-syn PFF-channel) over time in co-cultures. The intensity was taken as an index of the degree of accumulation within cells, with elevated or reduced intensity being proportional to increased/reduced aggregation from the previous 8 h bin. The ANOVA revealed a significant two-way (Time x Treatment) interaction for percent change in total frame fluorescence intensity of PFFs (F_5,24_ = 21.13, p < 0.001). Overall, the rate of 488-PFF signal change was clearly greatest during the first 16–24 h and levelled off to ∼0 (or no change) by 32 h (Fig. 2).

The changes in PFF fluorescence in the co-cultures were distinct depending upon which cell type was initially pre-treated with the PFFs. Specifically, the co-cultures prepared from PFF pretreated microglia showed a rapid reduction in overall PFF fluorescence within frames after 8 h in culture (compared to Time 0) and further significant reduction occurred during the next 8 h (p < 0.05, relative to Time 0). The rate of change in the PFF signal then became minimal by 16–24 h and relatively stable with little change by 32–48 h in the pretreated microglia co-cultures (Fig. 2). In contrast, co-cultures prepared from PFF pretreated neurons showed the opposite pattern, wherein a dramatic increase in α-syn PFF total frame area of fluorescence was evident during the first 8 h of being co-cultured with naïve microglia (p < 0.05, relative to later times and relative to the respective PFF_Mg groups). Then, during the subsequent 40 h, co-cultures from the pre-treated neurons progressively lost PFF signal, with maximal reductions evident around 32 h and then remaining fairly stable (Fig. 2). However, regardless of which cell was pretreated, it was clear that the PFFs can easily migrate from neuron to microglia and conversely, from microglia to neuron. Yet, exactly what is driving the migration is less obvious, but we did observe a clear change in cellular coverage area and fragmentation that is consistent with toxicity and disruption of membrane integrity.

### 3.2 PFF induced change in cellular morphology

**Fig. 3:**
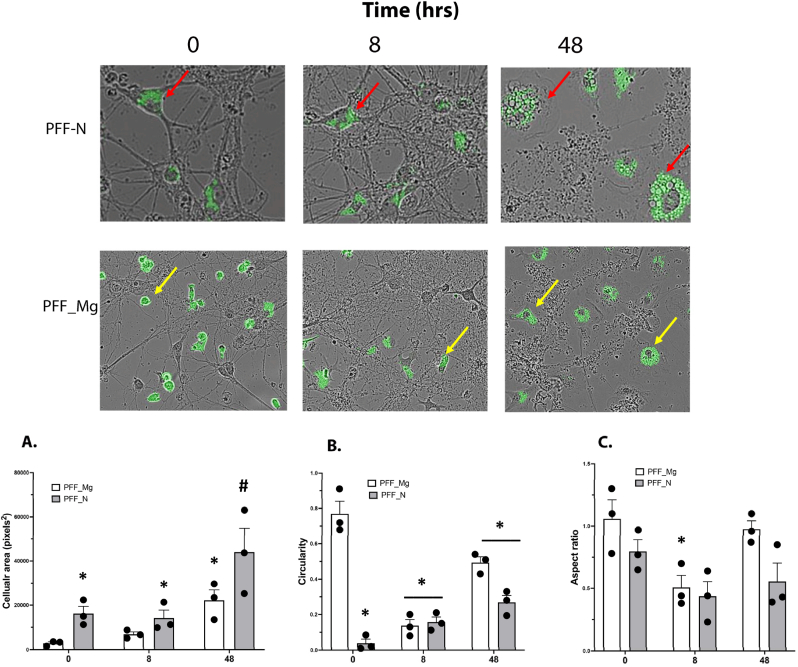
**Time course for total frame α-syn PFF fluorescence and morphological changes in primary neurons and microglia.** The top photomicrograph (PFF_N) depicts changes in PFF signal in co-cultures from PFF pretreated neurons after 0, 8 and 48 h. A profound cellular loss and breakdown of neural connections was observed by 48 h. An example of a PFF + neuron (red arrow) shows its structure beginning to break down as early as 8 h and the neuron was completely degenerated by 48 h. The remaining cells at 48hrs were enlarged foamy highly vacuolated microglia with robust PFF labelling, indicating extreme cellular distress. The co-cultures from PFF pretreated microglia (PFF_Mg) showed obvious changes in morphology by 8 h (yellow arrows). After 48 h, there were no longer any neurons visible and the remaining PFF + microglia were again mostly enlarged foamy cells with intracellular vacuoles present. The bottom bar graphs depict quantification of cell area, circularity and aspect ratio at the individual cell level. **(A)** The average area of PFF + cells was clearly increased in cultures from PFF_N pretreated neurons at all times (∗p < 0.05, relative to their respective PFF_Mg). At 48 h, the cell area was further increased for both PFF_Mg and PFF_N, ∗p < 0.05 and #p < 0.05, relative to the earlier times, respectively. **(B)** For circularity, the PFF_Mg pre-treatment clearly produced the highest level of circularity (with small rounded cells apparent) at the initial time point; but this was dramatically reduced over time (∗p < 0.05). In contrast, the PFF_N pre-treatment promoted an increase in circularity over time. **(C)** The aspect ratio (degree of elongation; width to height ratio) was significantly reduced only in the PFF-Mg group at 8 h, relative to the initial timepoint ∗(p < 0.05).

Ultimately, PFF levels in all treatment groups were the same by 48 h, at a time when there was obvious cellular degeneration observed (at least as suggested by time-dependent reductions in fluorescence cellular coverage area) and an obvious breakdown in neuronal structure. This was largely attributed to the obvious breakdown of neural connections and degeneration of the primary cortical neurons (Fig. 3). At this time, there were still many microglial cells present (albeit reduced compared to earlier times), but they all had very high PFF fluorescence. It appeared that the surviving microglial had taken up and accumulated the PFFs released from dying neurons. These microglia also had a highly “activated” morphology being large, with a flattened and swollen “fried-egg” appearance, and clearly different than those apparent at earlier time points. The microglia at this time also had many intracellular vacuoles present (Fig. 3), suggesting some degree of internal cellular distress and that they were likely in a highly pro-inflammatory state.

Quantification of cellular arear, circularity and aspect ratio of PFF + cells over time confirmed the obvious morphological changes observed. In terms of the area of individual cells, there were significant main effects for PFF pre-treatment (F_1,12_ = 10.82, p < 0.01) and time (F_2,12_ = 12.81, p < 0.001). Indeed, the comparisons indicated that PFF_N pre-treatment increase cellular area, relative to PFF_Mg exposure (∗p < 0.05), but that both PFF_N and PFF_Mg had further significant elevations at 48 h (∗p < 0.05 and #p < 0.05, relative to their respective earlier times; Fig. 3A). The overall pattern of results suggest increasing individual cell size over time, which we believe is likely fueled by the increasing presence of large swollen “foamy-like” microglia.

The degree of circularity of the individual cells was significantly altered between the groups, such that there was a significant PFF x Time treatment interaction (F_2,12_ = 43.1, p < 0.001). The Tukey's corrected follow up comparisons revealed that the degree of circularity in the PFF_Mg pre-treatment group at the initial time was significantly greater than any of the remaining times (∗p < 0.05, relative to later times; Fig. 3B). However, the circularity index for the PFF_N pre-treated cells significantly increased over time (∗p < 0.05, relative to earlier times), indicating progressively increasingly rounded morphology.

Finally, for the aspect ratio (or degree of elongation: width to height ratio), there was only a significant main effect for time (F_2,12_ = 7.90, p < 0.01), with the aspect ratio was significantly decreased at the 8 h time (∗p < 0.05, relative to the other times; Fig. 3C). This could possibly reflect a transitional cellular state or intermediate stage of cellular damage and microglial interaction with neurons.

### 3.3 PFF induced change in cellular coverage area

**Fig. 4:**
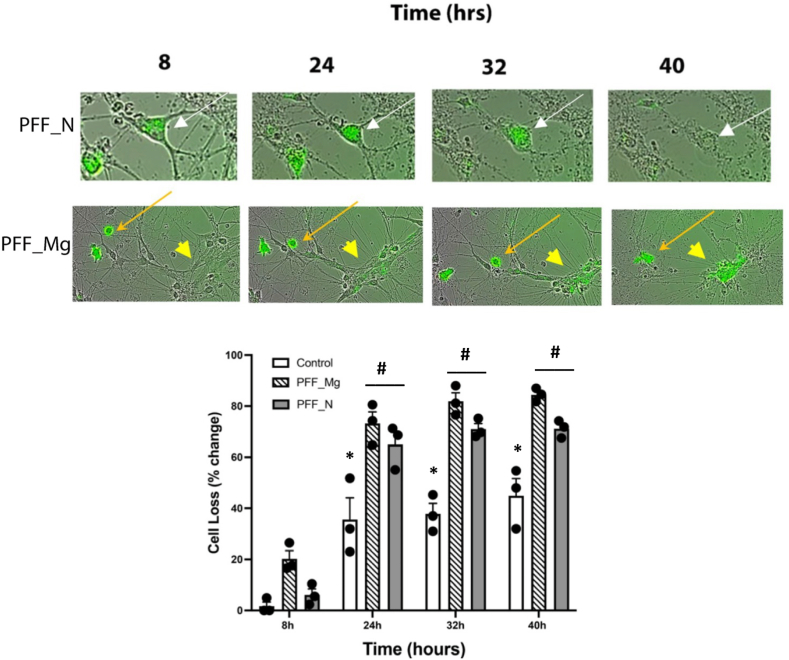
**Time-dependent change in cellular coverage area in PFF pre-treated microglia and neuron co-cultures.** The top photomicrographs depict PFF + labelling (green) of cells in co-cultures after 8–40 h of being placed together. Within co-cultures from PFF pretreated neurons (PFF_N), labelling is clearly observed in a neuron (arrow) and adjacent microglial cell (that accumulated the PFF after co-culture). Both cells were clearly degenerating by 32 h and were completely degraded by 40 h. In the co-cultures created from PFF pretreated microglia (PFF_Mg), the arrow clearly shows a PFF + rounded ameboid-like microglial cell and an adjacent cluster of naïve unlabelled neurons (large arrowhead). By 24–32 h the PFF + microglia (arrow) clearly migrated and made contact with the cluster of neurons. The neurons begin to accumulate PFF labeling by 24–32 h and by 40 h have largely degenerated with much diffuse labelling. The quantification of cellular loss is depicted in the bottom bar graph, with both neurons and microglia being appreciably lost over time [N = 3 biological replicates: (i.e. cells from separate litters). and 3–6 technical replicates (i.e. from different wells per condition/litter).]. Graph shows mean ± SEM. (∗p < 0.05, relative to 8 h time; #p < 0.05, relative to the control group).

Given the obvious signs of cellular degeneration we observed in the co-cultures, we next qualitatively and quantitively evaluated change in cellular coverage area and morphological changes. The PFF treatment did cause a significant change in cellular coverage area and this effect varied over time, as indicated by a significant PFF × Time interaction ((F_6,24_ = 2.72, p < 0.05). Indeed, both the PFF pretreated microglia and the PFF pretreated neurons caused a progressive change in cellular coverage area in their respective co-cultures (p < 0.05; Fig. 4). Specifically, PFF pre-treatment in both cell types eaually caused a reduction in cellular coverage area that was apparent by 24 h and still evident by 40 h upon their co-culture with the opposite and naïve cell type (#p < 0.05, relative to 8 h time and relative to the control treatment). The control (non-PFF) co-cultures also showed a significant reduction in cellular coverage by 24 h (∗p < 0.05, relative to 8 h), but this was significantly less than the PFF treated groups (#p < 0.05).

It was found that 488-PFF fluorescence could clearly move from one cell type to the other over time. This occurred prior to the breakdown of the PFF + seeded cell. Microglia typically moved into the vicinity of PFF + neurons and then appeared to interact and eventually engulf them. As shown in Fig. 3and Fig. 4, there was obvious disintegration of neuronal networks that was induced by the α-syn PFF pre-treatment that became apparent by 24 h. Then by 48 h the only apparent live cells were microglia with a greatly altered morphology. Indeed, heavily vacuolated fried egg-shaped cells indicative of highly activated microglia (most were PFF+) dotted the meshwork around the cellular aggregates.

### 3.4 PFFs time-dependently impact microglia morphologies

**Fig. 5:**
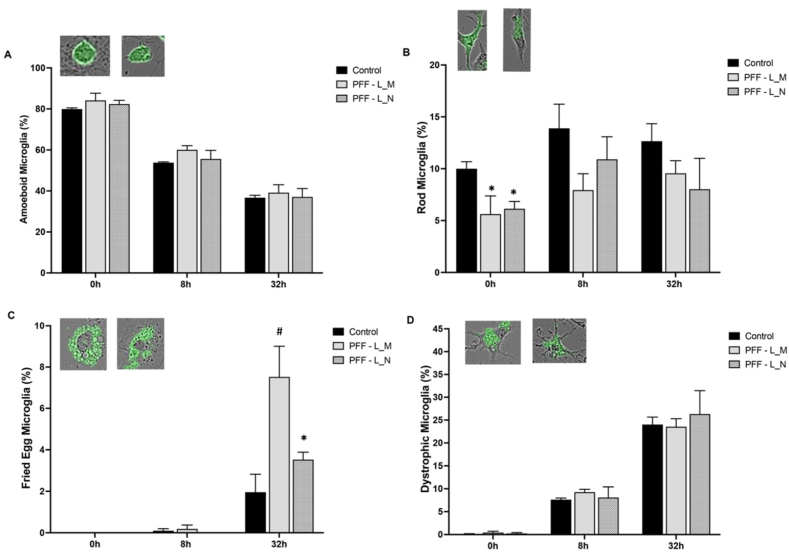
**Microglial phenotypic changes in co-cultures over time following PFF exposure.** We quantified the proportion of microglia displaying (A) Ameboid, (B) Rod, (C) Fried Egg and (D) dystrophic morphology. The bar graphs show that there was a reduction in ameboid type microglia over time in the co-cultures, but this was not significantly affected by PFF treatment. However, at the beginning of the co-culture (0 h), the percentage of rod-shaped microglia was significantly reduced by PFF pre-treatment in both labelled microglia (L_M) and labelled neurons (L_N). The fried egg foamy morphology was significantly increased by the microglial and neuronal PFF pre-treatment at 32 h, with microglial pre-treatment causing the largest increase. The number of dystrophic microglia increased over time, but not as a function of PFF pre-treatment. Two representative fluorescent PFF + images are given for the four predominate microglia morphologies observed in culture. ∗p < 0.05, relative to control; #p < 0.05, relative to PFF-L_N [N = 3 biological replicates: (i.e. cells from separate litters). and 3–6 technical replicates (i.e. from different wells per condition/litter).]. Graph shows mean ± SEM.

We next aimed to further characterize microglial responses and sub-types that were apparent over time in the co-cultures. Microglia were identified and categorized based on their morphological characteristics, which drastically changed over time with PFF exposure. The proportion of each distinct morphology present was assessed from all microglia observed at a particular time period. It should be noted that in the context of the present co-culture system, just simply adding the two cell types together (primary cortical microglia and neurons) creates somewhat of an “activated” phenotype and a pure *in vivo* ramified “resting” state was not observed in any cells. As shown in Fig. 5, the majority of microglia initially had an ameboid morphology, but this dramatically decreased over time. In contrast, other morphological phenotypes, such as microglia displaying an enlarged foamy fried egg morphology were only apparent at 32 h (in ∼10 % of cells) and the dystrophic type microglia were found to increase in percentage over time. The fried egg morphology was also associated with an excessive number of intracellular vacuoles, suggesting intracellular distress. In contrast, the rod-shaped microglia were reduced by the PFFs at the early, but not later times. We chose to focus upon these four different microglial morphologies (ameboid, fried egg, dystrophic and rod shaped) given that they were the most apparent and have had distinct characteristics.

With regards to the ameboid morphology, the PFFs had no significant impact at any time. However, the number of microglia exhibiting the ameboid shape was dramatically reduced over time in all groups (F_2,18_ = 188.7, p < 0.0001; Fig. 5A). The presence of dystrophic microglia was also unaffected by the PFF treatment, but increased progressively simply over time (F_2,18_ = 109.2, p < 0.0001; Fig. 5D). Hence, the ameboid and dystrophic phenotypes appear to follow a general inverse pattern over time.

The two remaining morphologies were significantly influenced by the PFF treatment (Fig. 5). Specifically, the number of rod-shaped microglia was significantly reduced at the earliest time point in co-cultures from PFF pretreated microglia or neurons (F_2,18_ = 5.3, p < 0.05; Fig. 5B). But there were no significant differences apparent at later time points. Finally, for the fried egg morphology there was a significant PFF × Time interaction (F_2,18_ = 8.4, p < 0.01; Fig. 5C). Specifically, the fried egg morphology did not occur at the earlier time points and only became evident by 32 h in the co-culture (∗p < 0.05, relative to earlier times). Interestingly, the PFF pretreated microglia caused the most robust increase in the percentage of microglia with this morphological subtype (#p < 0.05, relative to PFF-N group). Hence, the timing of the appearance of fried egg morphology appeared to most closely correlate with the cellular damage/loss of cellular area coverage that occurred over time.

### 3.5 PFF induced cytokine responses in PFF treated co-cultures

**Fig. 6:**
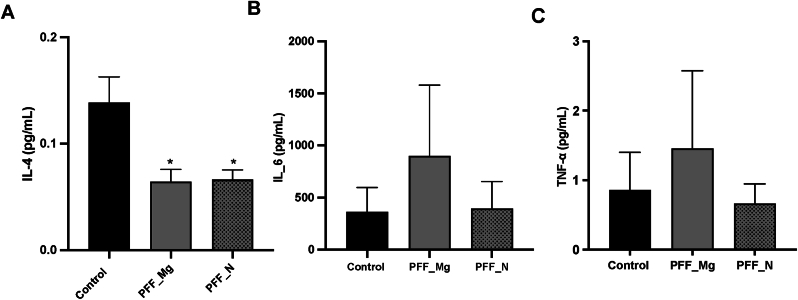
**Cytokine changes in microglia-neuron co-culture 48 h after pre-treatment of either neurons or microglia with PFFs.** Pre-treatment of either microglia (PFF_Mg) or neurons (PFF_N) with PFFs caused a significant reduction in extracellular IL-4 levels after 48 h in subsequent co-cultures (A). There was no impact of PFF treatment with regards to IL-6 (B) or TNF-a (C) levels in the media after 48 h ∗p < 0.05, relative to controls [N = 3 biological replicates: (i.e. cells from separate litters). and 3–6 technical replicates (i.e. from different wells per condition/litter).]. Graph shows mean ± SEM.

We finally sought to evaluate whether the morphological changes in microglia surviving PFF exposure are associated with a functional phenotypic shift in terms of their cytokines released. Accordingly, at the final 48-hr time, the extracellular levels of proinflammatory (IL-6, TNF-α) and anti-inflammatory (IL-4) cytokines were assessed in the media from the neuron-microglia co-cultures. The ANOVAs for IL-6 and TNF-α failed to reveal any significant differences between groups. However, a significant difference in IL-4 levels across treatment groups was apparent (F_2,4_ = 5.45, p < 0.05). As shown in Fig. 6 and confirmed by the follow up comparisons, the levels of IL-4 were significantly decreased in all cultures that were exposed to the PFFs (both microglia and neuron pretreated) (p < 0.05). Hence, the PFFs may be able to reduce at least some of the anti-inflammatory signaling capacity of the surviving cells.

## 4 Discussion

The misfolded fibril form of the α-syn protein is a potent toxic factor that can directly or indirectly influence neuronal survival [12,18]. In addition to its accumulation in neurons, microglial cells may also take up oligomeric and fibril forms of α-syn [19,20]. But it is unclear as to how seeding of a particular cell type impacts subsequent neuron-microglial interactions. Hence, the present study separately exposed either primary neurons or microglia to 488-labelled α-syn PFFs, allowing for their uptake and aggregation, before then co-culturing these cells with the opposite treatment naïve cell type. This allowed us to assess the behavior of naïve and seeded cells together and whether the α-syn PFFs spread from one cell type to another and impact neuronal survival. We found that the PFFs were indeed readily taken up individually by both neurons and microglia and that upon subsequent co-culture, caused obvious changes in microglial and neuronal morphology, eventually leading to signs of cellular damage. This co-culture system is far more intricate than simply an individual neuron or microglial mono-culture and allows for a controlled and direct experimental manipulation of each separate cell type prior to their integration into the co-culture.

### 4.1 Α-syn PFF total frame fluorescence intensity and toxicity

*In vivo* α-syn PFF models are generally thought to produce an approximation of the pathological accumulation observed in PD [2,[21], [22], [23]] and may also be relevant to other synucleinopathies (including Lewy body dementia) [2,23]. *In vitro* PFF models allow for more direct manipulation of specific cell types and it has been suggested that intracellular α-syn inclusions in primary neuron cultures may resemble the pathology evident in the early stages of PD [24,25] and hence, may be useful for testing aggregation-preventative treatments [2,3]. Our analyses clearly showed that PFF exposure induced dynamic time-dependent changes in neuronal and microglial migration and interactions.

Using live cell imaging, we found that co-cultures containing PFF pre-treated microglia (together with untreated neurons) displayed rapid reductions in the accumulation of 488-labelled α-syn over time, whereas co-cultures that had PFF pre-treated neurons (together with untreated microglia) showed an early increase followed by a progressive decrease in PFF accumulation. In the former case, the PFF pre-treated microglia co-cultures resulted in a spread of the labelled PFFs from microglial into neurons after several hours and their subsequent signs of cellular damage within 24–48 h. Hence, the PFF seeded microglia appeared to be migrating to neurons and causing adverse effects over time. In the latter case when neurons were pretreated with PFFs, it was clear that microglia were accumulating α-syn PFFs released from progressively damaged or morphologically compromised neurons. This suggests that the neuronal endangerment provoked by PFF uptake likely resulted in a signal being relayed to promote microglia to adopt a phagocytotic or other activated state that was aimed at encapsulating free PFFs.

PFF uptake by microglia from damaged or morphologically altered neurons may represent an adaptive response designed to protect neurons. Yet, when PFF seeded microglia were added to naïve neurons they still resulted in cellular area changes consistent with damage and eventually neuron loss. This suggests that the PFFs could induce a neurotoxic phenotype in microglia. So, the end result was the same regardless of the initial PFF seeded cell type, but the timing and intermediate mechanistic steps may differ. It is however, important to note that the co-culture method alone was associated with some change in cellular coverage area over time, due to the fact that primary microglia were added to a pre-established primary neuronal culture resulting in disrupted homeostasis.

One other study, which was done *in vivo*, reported that α-syn inclusions first occurred in neurons and then secondly to glial cells, primarily astrocytes, in a genetic α-syn expressing model [26]. It was interesting that neurons were also more vulnerable to α-syn toxicity, dying much earlier and to a greater extent than astrocytes [26]. It was posited that the delayed time for astrocyte accumulation of α-syn aggregates might reflect the phagocytic factions of the astrocyte in the face of neuronal damage and debris. This could also certainly be the case in the present situation. Indeed, microglia were observed to migrate to α-syn seeded neurons and clear signs of cellular damage/distress were present; most notably large vacuoles and abnormal morphology. Likewise, we also show that PFF pre-treatment of microglia alone caused their uptake of labelled fibrils and that these primed microglia reduced cellular area fluorescence upon their subsequent co-culture with naïve neurons.

We observed obvious vacuolation in the few cells that were visible 48 h after PFF exposure. Autophagic vacuole induction is classical protective process that attempts to elicit lysosomal elimination of intracellular damaged or pathological proteins, but this process can also promote cellular death [27]. Indeed, vacuolation in the cytoplasm is a primary characteristic of membrane breakdown and necrotic death. Yet, autophagy is also a fundamental means of breakdown of α-syn rich Lewy bodies [28,29], but α-syn overexpression can eventually overcome cellular resources and impair autophagy [30]. In fact, neuronal α-syn was reported to activate microglia, promoting their capture of α-syn into autophagosomes for degradation [19]. Along these lines, vacuolation may reflect ongoing autophagic degradation of α-syn PFFs, along with damaged cellular material.

Of course, there is the possibility that any present vacuoles are not necessarily tied to autophagy, but rather to certain microglial phenotypic shifts. In fact, a re-distribution of lipids in microglia exposed to lipid-nanoparticles elicited a highly vacuolated foamy microglial phenotype [31]. These vacuolated microglia have been referred to as, Gitter cells, and are enlarged with a distended cytoplasm, similar to the present cells we observed 48 h after α-syn exposure. This state has been linked to overwhelmed phagocytosis following excessive interactions with infectious agents or cellular debris. It is reasonable to posit that excessive phagocytosis of α-syn PFFs and neuronal debris presently observed may have placed microglia in such an exhausted state.

### 4.2 Impact of α-syn on microglial phenotype and interactions

Microglia perform constant surveillance and engage in crosstalk with neurons in order to orchestrate appropriate signaling upon encountering any immune or toxicological insult [32,33]. Communication is bidirectionally mediated by a variety of factors including purines, toll-like receptors (TLRs) and cytokines that modulate cell motility neuron-microglia signalling [34]. It has recently been reported that α-SYN fibrils can be rescued from seeded neurons by microglial through tunneling nanotubes (Scheiblich et al., 2024). However, it is unclear as to whether α-SYN may migrate from microglia to neurons by these nanotubes.

Much work has suggested that neurons accumulate α-syn through a variety of mechanisms, including endocytosis, that facilitate its intercellular spread and prion-like propagation. Yet, it is unclear as to exactly how microglia might accumulate α-syn. Of course, a primary route may be by phagocytosis of the misfolded protein, which could occur through pattern recognition receptors, such as the TLRs. For instance, microglial TLR2 or TLR4 may sense extracellular α-syn and trigger NF-κB signaling to upregulate p62/SQSTM1, thereby promoting autophagic degradation of internalized α-syn [19]. But, it has also been reported that the Mer tyrosine kinase (MerTK) receptor can facilitate non-inflammatory phagocytosis of α-syn fibrils by human microglia [35]. Whatever the case, we presently show that primary murine microglia respond to fibril α-syn with marked morphological changes that subsequently have adverse consequences on co-cultured primary cortical neurons. It remains to be determined whether PFFs might be extracellularly released from activated microglia to impact neurons.

Microglia are also phenotypically diverse and while often broadly characterized in an overly simplistic manner as ‘M1’ pro- and ‘M2’ anti-inflammatory, in reality these cells exist in a wide range of differing “reaction or activation” states in between these two extremes (Ceccatelli et al., 1991; [36,37]. While the classic M1/M2 phenotypes are not perfectly aligned with the various stages of microglial responses in neurodegenerative disease, they still serve as useful basic guideposts [38,39].

During the change in cellular coverage area that was presently observed to progress over the 48-hrs in co-cultures, the microglia adopted morphologies that generally allowed for increased area coverage and were indicative of “activated” phenotypic states. These changes allow for enhanced microglial surveillance and involve mobilization of actin-dependent filopodia and large process that are linked to purinergic and cAMP signaling [40]. Indeed, the microglial changes are rapid and dramatic following an inflammatory insult or injury [41,42] and can display (among other variations): 1. thickened processes and elongated cell bodies (hypertrophic/dystrophic), or 2. enlarged cell bodies and retracted processes (ameboid), or 3. long thin cell bodies with short and slender processes (rod-like) or 4. large, round cell body with no processes and contain cell debris (fried egg). We presently identified microglia displaying each of these four morphological categories and many times, these morphological states were also associated with the presence of intracellular vacuoles. These vacuoles are a general indication of cellular distress and that autophagic processes (degradation of cellular debris or protein fragments) are likely ongoing. It should be noted that because of the nature of our co-culture system we did not find any purely ramified (which *in vivo* is a reflection of a typical “resting” or non-activated state) microglial cells at any time point. This is not uncommon and in fact, is a general caveat of cell culture work. That said, our live cell imaging allowed us to capture a range of highly dynamic activated morphologies.

The multiple microglial morphological categories varied greatly over time and with PFF treatment. All groups initially had many ameboid microglial cells, as was reported previously [43], but these decreased over time as the other morphological phenotypes emerged. In contrast, the rod shape microglial type was present in similar numbers over time, which is consistent with previous findings, indicating these microglia are typically stable [44]. A fried egg microglial morphology was only significantly present in co-cultures at 32h, especially in these cultures that contained PFF pre-treated microglia. This is consistent with a previous study that showed that α-syn dose-dependently increased the fraction of activated primary microglial mono-culture with a “fried egg” appearance [45]. The fried egg shape was also recently characterized following bacterial insults or following LPS + IFN-γ administration [44,46]. These data overall suggest that the PFFs had obvious effects upon the morphology of microglia over time.

### 4.3 Impact of α-syn on secreted cytokines

In addition to morphology, the cytokines produced by microglia are an important indicator of their phenotype. The more pro-inflammatory type microglia characteristically produce the cytokines, IL-1β, TNF-α, IL-6, whereas microglia of an anti-inflammatory phenotype express IL-10, IL-4 and Arg1, with, in between phenotypes less well characterized [36]. Surprisingly, we found the α-syn treatments had no impact on the levels of pro-inflammatory cytokines, IL-6 and TNF-α. However, a major limitation of this study is the fact that cytokine levels were only measured at the final time point (after 48 h), at which time extensive cellular degeneration is observed and it is highly likely, that most pro-inflammatory changes occurred much earlier.

We did observe an IL-4 reduction in co-cultures containing PFF pre-treated cells (either microglia or neurons), which might reflect an enhanced proinflammatory microglial state, since IL-4 is anti-inflammatory and can exert beneficial effects on neuronal survival in animal models of PD [47,48]. This is also intriguing in light of studies that have reported that IL-4 can drive an “M2”-like microglial phenotype that was critical for recovery after cerebral ischemia [49,50]. It is also pertinent that IL-4 can increase the phagocytotic activity of cultured microglia [51], raising the possibility that this cytokine played a role in prompting microglia to degrade α-syn PFFs. Yet, the role of IL-4 is undoubtedly complex since one study involving central LPS infusion found that IL-4 actually appeared to contribute to the death of dopamine neurons [52].

### 4.4 Limitations and conclusions

It should be noted that a major limitation of the present data is the fact that the imaging method used does not distinguish between surface-bound and internalized PFF signal. Hence, we cannot be certain whether or not the observed PFF fluorescence reflects uptake or internalization. Likewise, we did not directly characterize the PFF preparations at a biophysical levels, using TEM or other high magnification imaging. The interpretations of their effects are therefore based on the preparation protocol and imaging data alone. Finally, since the co-culture model does not separate contact-dependent from soluble-factor-mediated effects, the results should be interpreted with this design limitation in mind.

With these caveats in mind, the present co-culture system did utilize a highly novel approach to address how pre-treating one cell type (microglia vs neuron) with a toxic (α-syn) seeding factor influences interactions when then co-cultured with the opposite naïve (untreated) cell type. We found that α-syn PFF pre-treatment of either cell type ultimately resulted in similar outcomes, reflected in microglial morphologies and loss of cellular fluorescence area coverage (as an index of possible neuronal damage/degeneration). It is of course, important to note that microglia-neuron communication is also influenced by astrocytes, as well as any infiltrating immune cells [34,53]. These cells would add to the complexity of the *in vivo* situation beyond that presently observed. Nonetheless, we believe our approach provides a temporally sensitive means to better understand real time α-syn trafficking between microglia and neurons and the ensuing toxicity.

## CRediT authorship contribution statement

**C. Paquette:** Investigation, Methodology, Writing – original draft. **T. Charlton:** Data curation. **N. Prowse:** Methodology. **T. Fortin:** Methodology. **H. Sun:** Funding acquisition, Investigation. **S. Hayley:** Conceptualization, Data curation, Formal analysis, Funding acquisition, Resources, Supervision, Visualization, Writing – review & editing.

## Declaration of competing interest

The authors declare that they have no known competing financial interests or personal relationships that could have appeared to influence the work reported in this paper.

## Data Availability

Data will be made available on request.
